# A giant partial thrombosed aneurysm of the internal cavernous carotid artery mimicking a meningioma of the lesser wing of the sphenoid bone

**DOI:** 10.1016/j.radcr.2022.01.075

**Published:** 2022-02-19

**Authors:** Mehdi Borni, Fatma Kolsi, Ines cherif, Mohamed Zaher Boudawara

**Affiliations:** Department of Neurosurgery, UHC Habib Bourguiba, Sfax, Tunisia

**Keywords:** Giant aneurysm, Meningioma, MRI, Angiography

## Abstract

Giant intracranial aneurysms are defined as those with diameters of 25 mm or more and represent about 5% of all intracranial aneurysms. These aneurysms typically manifest during the fifth to seventh decades of life. Due to their size, giant aneurysms are responsible for intracranial mass effect rather than hemorrhage. Clinical symptoms depend on aneurysm's location. Radiological features are not common for aneurysms of the internal cavernous carotid artery. Differential diagnosis includes pituitary adenoma, meningioma, craniopharyngioma, hamartoma, glioma, teratoma, and even granuloma.

Here, the authors report a case of a 63-year-old female patient with a giant partial thrombosed aneurysm of the internal cavernous carotid artery mimicking a meningioma of the lesser wing of the sphenoid bone who presented for visual defect, and raised intracranial pressure. The authors will proceed with a literature review investigating this entity as well its ability of mimicking meningioma.

## Introduction

Giant intracranial aneurysms have always been, and remain, among the most difficult cerebrovascular lesions to treat. They are defined as those with diameters of 25 mm or more and represent about 5% of all intracranial aneurysms [1,2]. These aneurysms typically manifest during the fifth to seventh decades of life and have a significant female predominance [3]. Due to its size, a giant aneurysm is responsible for intracranial mass effect rather than intracranial hemorrhage. Clinical symptoms depend on aneurysm location [4,6]. Radiological features are not common for aneurysms of the internal cavernous carotid artery. Differential diagnosis includes pituitary adenoma, meningioma, craniopharyngioma, hamartoma, glioma, teratoma, and even granuloma.

Here, the authors report a case of a 63-year-old female patient with a giant partial thrombosed aneurysm of the internal cavernous carotid artery mimicking a meningioma of the lesser wing of the sphenoid bone who presented for visual defects in both eyes, and raised intracranial pressure. The authors will proceed with a literature review investigating this entity as well its ability at mimicking meningioma.

## Case report

**Fig. 1:**
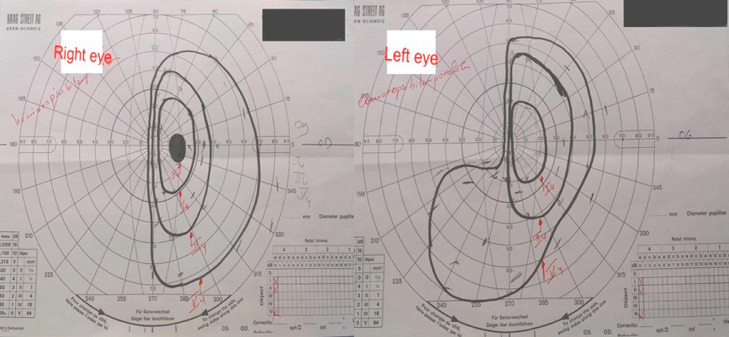
Goldmann visual field perimetry showing bitemporal hemianopsia.

**Fig. 2:**
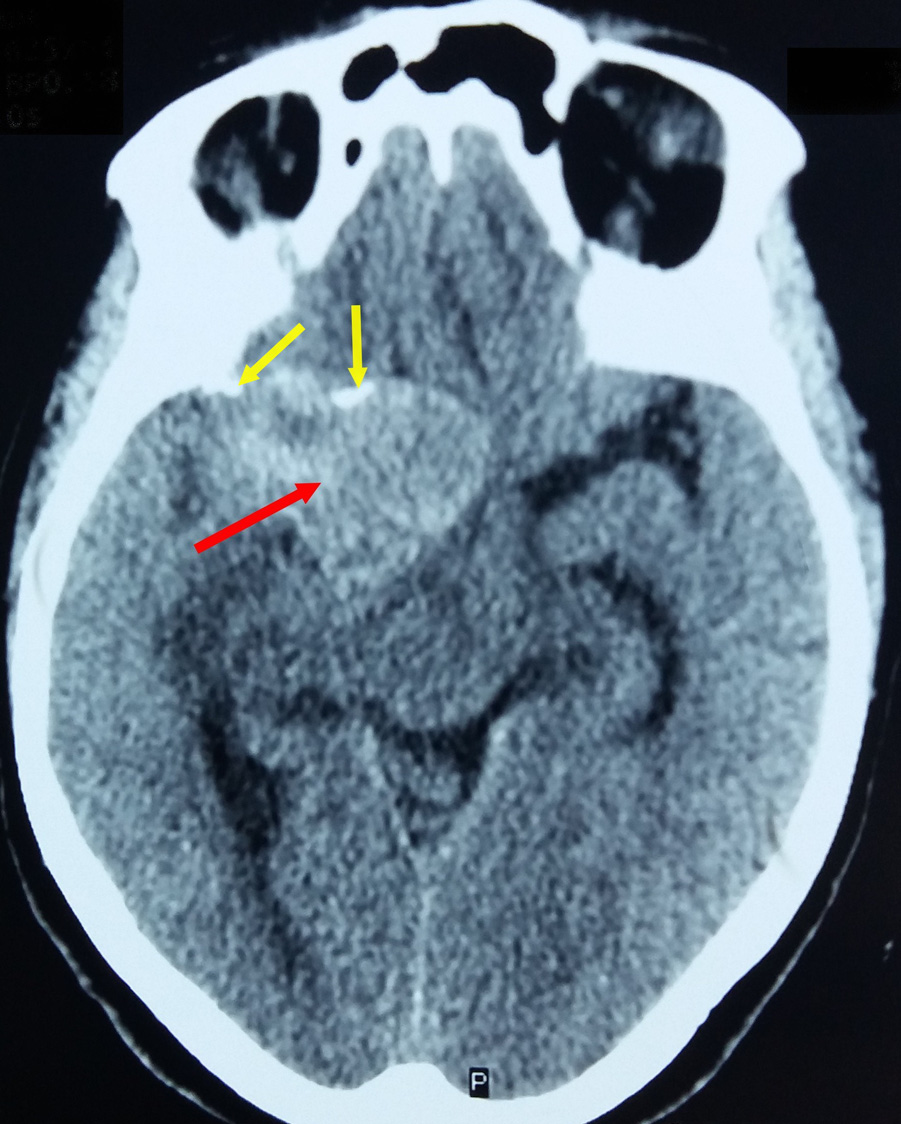
Axial non–enhanced computed tomography (CT) scan in parenchymal window showing a right extraaxial anterior temporal parasellar lesion (red arrow) probably originating from the anterior clinoid process with cranial extension holding calcifications (yellow arrows). The whole was surrounded by perilesional edema resulting in a subfalcine herniation and mass effect on the brainstem (Color version of the figure is available online.)

A 63-year-old female patient with no medical or surgical history who was referred by the basic health unit of her locality to our department of Neurosurgery for 1-year history of recurrent visual defects in both eyes associated to a right temporal throbbing pulsating headache and left body heaviness with progressive onset. There was no clinical history of vomiting or seizures. The patient was awake, communicative, and well oriented in time and space on neurological examination. A decreased visual acuity was observed in both eyes and a bitemporal hemianopsia was confirmed by the Goldmann visual field perimetry (Fig. 1). The patient had a right relative afferent papillary defect and mild sensory loss in the left ophthalmic nerve distribution. There was a significant grade 3 hemiparesis associated to a grade 3 facial palsy according to House and Brackman's classification. A first non–enhanced computed tomography (CT) scan (Fig. 2) was performed showing a right extraaxial anterior temporal parasellar lesion probably originating from the anterior clinoid process of the sphenoid bone with cranial extension holding calcifications. The whole was surrounded by perilesional edema resulting in a subfalcine herniation and mass effect on the brainstem. All these features suggested a meningioma originating from the lesser wing of the sphenoid bone.

**Fig. 3:**
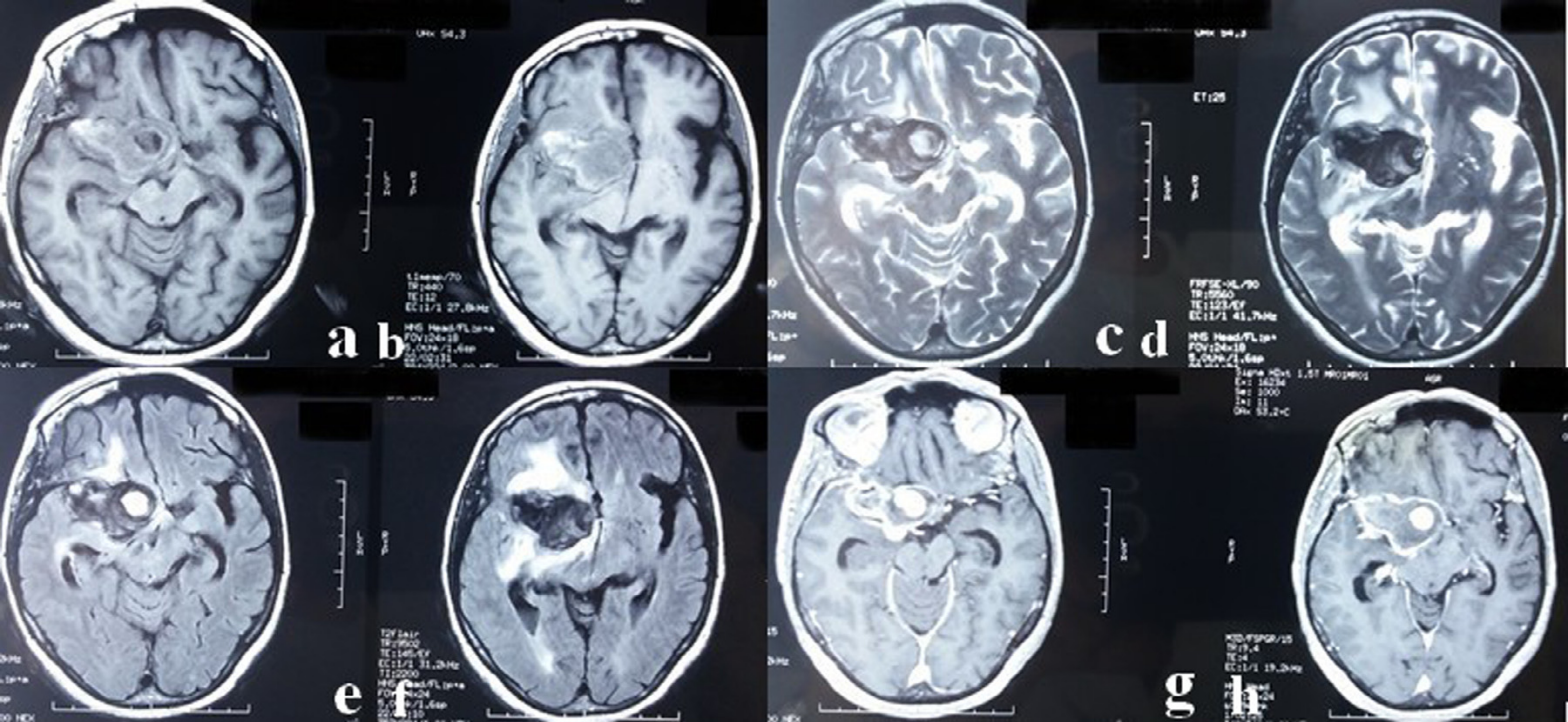
Axial brain magnetic resonance imaging (MRI) showing a suprasellar lesion measuring 45 × 4 × 39 in diameter lateralized to the right side with heterogeneous hyposignal on T1 weighted image (A,B) and hyposignal on T2 weighted image (C,D). The lesion appeared also in heterogeneous hypersignal on the Fluid attenuated inversion recovery (FLAIR) sequence (E,F) with a significant surrounding perilesional edematous reaction resulting in a mass effect on the optic chiasm and the third ventricle as well as an active biventricular hydrocephalus. Note the peripheral heterogeneous enhancement similar to the arterial network communicating with the internal cavernous carotid artery after Gadolinium administration (G,H) suggesting a partial thrombosed aneurysm.

**Fig. 4:**
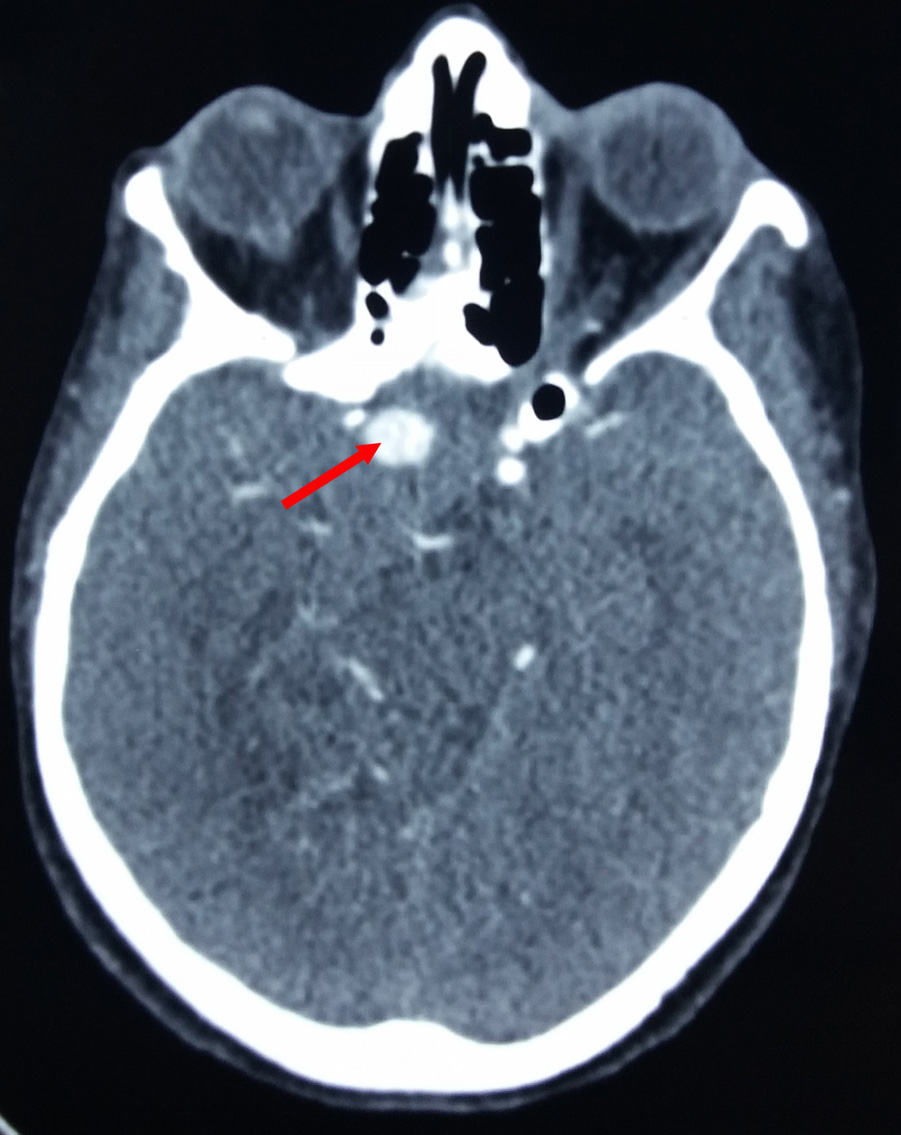
CT angiography showing the saccular aneurysm (red arrow) that measured 1.7 cm in maximal dimension. Enhancement was similar to the arterial network and there was a significant communication with the internal cavernous carotid artery. No additional aneurysms were identified and there was no arterial occlusion or hemodynamically significant narrowing (Color version of the figure is available online.)

Subsequently, our patient was submitted to a brain magnetic resonance imaging (MRI) (Fig. 3) revealing a suprasellar lesion measuring 45 × 4 × 39 in diameter lateralized to the right side with heterogeneous hyposignal on T1 weighted image and hyposignal on T2 weighted image. The lesion appeared also in heterogeneous hypersignal on the Fluid attenuated inversion recovery (FLAIR) sequence with a significant surrounding perilesional edematous reaction resulting in a mass effect on the optic chiasm and the third ventricle as well as an active biventricular hydrocephalus. After Gadolinium administration, there was a peripheral heterogeneous enhancement similar to the arterial network communicating with the internal cavernous carotid artery suggesting a partial thrombosed aneurysm. Further examination by a CT angiography (Fig. 4) was performed. A saccular aneurysm that measured 1.7 cm in maximal dimension was then showed. Enhancement was similar to the arterial network and communicating with the internal cavernous carotid artery. No additional aneurysms were identified and there was no arterial occlusion or hemodynamically significant narrowing.

A selective conventional angiography of the supra-aortic vessels in supine position, under general anesthesia was indicated but the patient refused any further investigation, and was then discharged from hospital against medical advice.

## Discussion

As it had been said, giant aneurysms are defined as those with diameters of 25 mm or more. They represent about 5% of all intracranial aneurysms [1,2].  These aneurysms typically manifest during the fifth to seventh decades of life with a significant female predominance [3]. They were defined in 1977 by Segal and McLaurin as partially thrombosed with residual tortuous vascular channels [5]. Most giant aneurysms are of the saccular type [6].

Microscopic studies demonstrate that the aneurysmal wall consists of fibrous tissue measuring 2-4 mm in thickness often with calcification, loss or partial defect of the internal elastic lamina and media. Aneurysmal sac contains thrombi of various ages with calcifications and hemorrhage [4] as it was seen in our case. The intra-aneurysmal vascular channels do not seem to be residual lumens of the parent artery but are typically intrathrombotic canals that are not endothelialized and do not contain normal elastic lamina or media. The canals may be central or eccentric, with typical slow flow [7,8].

According to the international study of unruptured intracranial aneurysms, giant aneurysms carry a 6% annual risk of rupture compared with a 1%-3% annual risk for smaller aneurysms [9]. Some reports indicate that these risks may be even greater. It has been reported that over 50% of giant aneurysms will rupture [6]. Mortality rates in patients with untreated giant aneurysms have been reported to be 68% and 80% at 2 and 5 years, respectively [6,10]. Our patient did not present any sudden neurological symptoms related to the rupture of her aneurysm but rather it was a syndrome of raised intracranial pressure and visual defects that led her to consult us.

Clinically, patients with giant aneurysms usually suffer from classic intracranial mass effect rather than intracranial hemorrhage. Clinical symptoms depend on aneurysm's location [4]. The neurological clinical signs of our patient were related to the parasellar localization occupying the middle temporal cerebral fossa thus compressing the visual anatomic structures as well as the midbrain. All this was responsible for a contralateral hemiparesis and a visual defect. Large and giant aneurysms of the cavernous, ophthalmic, and hypophyseal segments of the internal carotid artery frequently present with symptoms of mass effect on the cranial nerves with diplopia in 1 or multiple direction of gaze with or without ptosis and pupillary dysfunction. Compression of the oculomotor nerve, trochlear nerve, or abducens nerve results in ophthalmoplegia, and it is frequently associated with cavernous sinus aneurysms as it was described in our current case. Optic nerve compression results in decreased visual acuity and visual field deficits and is mostly associated with carotid ophthalmic or superior hypophyseal artery aneurysms [11]. Some cases may be totally asymptomatic. In 2018, Packer et al. [12] reported a case of a 69-year-old lady who was admitted after a traumatic brain injury. She was completely asymptomatic and carried with her a brain CT scan, performed 24 hours after the injury, showing left nodular parasellar lesion involving the cavernous sinus, discovered at random.

Imaging findings of these giant aneurysms are characteristic. At unenhanced CT scan, they appear as well-delineated, round or lobulated, slightly hyper attenuating extra axial masses, often with peripheral intramural or luminal thrombus calcification as it was described in our patient's scan. Significant mass effect may be evident as it was seen in our patient. The signal intensity characteristics at MRI are variable and depend on blood velocity and turbulence as well as the age of intraluminal thrombus. Concentric bands of thrombus within giant aneurysms commonly produce a heterogeneous, laminated appearance [6]. Conventional angiography or CT or MR angiography is essential to assess intraluminal flow, define the aneurysm neck, and establish the precise relationship of the aneurysm to adjacent arterial structures [3].

Differential diagnosis of sellar and parasellar lesions includes, in addition to pituitary adenomas and aneurysms, other neoplasms such as meningioma, craniopharyngioma, hamartoma, glioma, teratoma, and even granuloma. The diagnostic arguments for intrasellar aneurysms, according to Guiot [13], would be the sudden occurrence of visual defect, with frequent unilateral blindness, severe headache, and a hyperdense lesion on CT scans. Daniels et al. [14] added that at CT scan, a rim of calcification is more consistent with aneurysms as it was seen in our case, while focal calcification suggests craniopharyngioma or meningioma, and that aneurysms are more eccentric than other sellar masses.

Management of giant intracranial aneurysms is challenging due to their complex anatomic features [3,6,10]. Optimal therapy depends on aneurysm size, shape, relation to adjacent neural anatomic structures, and the presence of collateral circulation or intraluminal thrombus [6]. Endovascular therapy (including embolization with or without stent or balloon assistance) may be used alone or in combination with surgical techniques [8]. When possible, direct clip occlusion is usually the preferred surgical technique [3]. Alternative surgical techniques include parent vessel occlusion, aneurysm trapping, aneurysmectomy, and aneurysmorrhaphy [3,10]. A preoperative balloon occlusion test may be performed to determine if patient will tolerate sacrifice of parent arteries. In patients with poor collateral circulation, surgical bypass may be a necessary therapeutic component [3]. In our patient we did not, unfortunately, perform any of these therapies as she decided to be discharged against our medical advice.

The rupture of these aneurysms is a catastrophic event, and most patients who survive have severe neurologic dysfunction [3]. Ever-evolving technologies continue to broaden therapeutic options for patients with giant intracranial aneurysms and to improve morbidity and mortality.

## Conclusion

Giant aneurysms of the internal cavernous carotid artery are clinically manifested by headache and sudden complex ophthalmoplegia associated or not with other signs and symptoms. Cranial X-rays show saddle alterations that may lead to misdiagnosis of other pathologies in this region. The most sophisticated radiological exams (CT and MRI) are often not enough to make the differential diagnosis of lesions in this region, requiring further cerebral angiography. Surgical treatment, by direct clip occlusion is usually the preferred surgical technique in correcting the symptoms of these patients, and has a low rate of complications.

## Patient consent

Written informed consent was obtained from the patient for publication of this case report and any accompanying images.

## Authors' contributions

All authors contribute to conception and design, acquisition of data and its analysis and interpretation. All authors were included in the drafting the article and its revision critically for important intellectual content. All authors finally approve the version to be published.
